# Retraction: MiR-205 suppresses epithelial-mesenchymal transition and inhibits tumor growth of human glioma through downregulation of HOXD9

**DOI:** 10.1042/BSR-2018-1989_RET

**Published:** 2024-07-03

**Keywords:** Epithelial-mesenchymal transition, Glioma, HOXD9, miR-205, tumor

This article is being retracted from *Bioscience Reports* at the request of the Editor-in-Chief and the Editorial Board following receipt of a notification from a reader, alerting the Editorial Board to concerns surrounding the appearance of Western blots in this paper, and an image in this paper that seems to appear in another article. The Western blots appear to have minimal background details and share similarities with other Western blots present in papers by different authors. Additionally, Figure 2D U87 miR-NC panel seems to appear as Figure 2E H1299 miR-33b inhibitor panel from Zhai et al, 2019 (DOI: 10.1002/jcb.27961) with rotation. The authors have been contacted with regards to the retraction and have not responded to the Journal's queries or the concerns raised. Given the extent of the issues raised, the Editorial Board stand by the decision to retract the article.

